# In Silico Prediction of the Toxicity of Nitroaromatic Compounds: Application of Ensemble Learning QSAR Approach

**DOI:** 10.3390/toxics10120746

**Published:** 2022-12-01

**Authors:** Amirreza Daghighi, Gerardo M. Casanola-Martin, Troy Timmerman, Dejan Milenković, Bono Lučić, Bakhtiyor Rasulev

**Affiliations:** 1 Biomedical Engineering Program, North Dakota State University, Fargo, ND 58105, USA; 2 Department of Coatings and Polymeric Materials, North Dakota State University, Fargo, ND 58102, USA; 3 Department of Computer Science, North Dakota State University, Fargo, ND 58105, USA; 4 Department of Science, Institute for Information Technologies, University of Kragujevac, 34000 Kragujevac, Serbia; 5 NMR Centre, Ruđer Bošković Institute, 10000 Zagreb, Croatia

**Keywords:** toxicity, nitroaromatic compounds, QSAR, QSTR, machine learning, Accumulated Local Effect, support vector machine, ensemble model

## Abstract

In this work, a dataset of more than 200 nitroaromatic compounds is used to develop Quantitative Structure–Activity Relationship (QSAR) models for the estimation of in vivo toxicity based on 50% lethal dose to rats (LD_50_). An initial set of 4885 molecular descriptors was generated and applied to build Support Vector Regression (SVR) models. The best two SVR models, SVR_A and SVR_B, were selected to build an Ensemble Model by means of Multiple Linear Regression (MLR). The obtained Ensemble Model showed improved performance over the base SVR models in the training set (*R*^2^ = 0.88), validation set (*R*^2^ = 0.95), and true external test set (*R*^2^ = 0.92). The models were also internally validated by 5-fold cross-validation and Y-scrambling experiments, showing that the models have high levels of goodness-of-fit, robustness and predictivity. The contribution of descriptors to the toxicity in the models was assessed using the Accumulated Local Effect (ALE) technique. The proposed approach provides an important tool to assess toxicity of nitroaromatic compounds, based on the ensemble QSAR model and the structural relationship to toxicity by analyzed contribution of the involved descriptors.

## 1. Introduction

Nitroaromatic compounds (NACs) belong to the largest group of industrial chemicals that exhibit various aspects of toxicity such as immunotoxicity, skin sensitization, germ cell degeneration, mutagenicity, and carcinogenicity [1,2]. Pesticides, explosives, drugs, cosmetics, herbicides, antioxidants, gasoline additives and corrosion inhibitors are good examples of the use of NACs in industry [3,4,5]. These compounds are almost exclusively released to the environment from industrial activities and anthropogenic sources. Studies focusing on this discovered that NACs are widely distributed in the biosphere and cause serious pollution in water, soil, atmosphere as well as food via absorption and bioaccumulation in the food chain [3,6]. The main toxicity behavior of NACs is described as uncoupling agents in oxidative phosphorylation [7,8]. Other toxic effects were reported for NACs in relation to the formation of various types of high-level contaminants and hazardous compounds such as nitropyrene, nitronaphthalenes, nitrofluorenes, 3-nitrobenzanthrone, nitroanthracenes and nitrophenanthrenes [9,10].

Quantitative Structure–Activity Relationship (QSAR) is used for the last three decades as a reliable tool for a multifaceted study of the toxicity of chemicals from various aspects [10,11,12,13,14,15]. In general, QSAR modeling attempts to link structure of compounds with their biological activities, physiochemical and toxicological properties to provide reliable predictive information based on experimental data [16,17].

In recent years, NACs are still of great interest to various research groups that mainly focused on environmental toxicology [18,19,20,21,22]. Many of these studies relate to the use of QSAR techniques to investigate the aquatic toxicity of NACs [15,23,24]. On the other hand, there are only a few papers dealing with the oral toxicity of NACs to animal level [9,11,25].

In 2008, Kuzmin et al., [26] published a QSAR model based on the simple representation of molecular structure (SiRMS) approach. In this study, a dataset of 28 NACs was used to generate the 1D−2D indices for predicting in vivo oral acute toxicity (rats) in terms of LD_50_. The partial least square 2D QSARs showed reasonable performance values with *R*^2^ = 0.96–0.98 for the training set and *R*^2^ = 0.89–0.92 for the test set. These authors also showed that hydrophobicity, electrostatic and Van der Waals interactions, and the addition of hydroxyl (-OH) and fluorine (H_2_F and CH_2_F) groups contribute to the enhancement of toxicity, while the introduction of methyl groups leads to a decrease in toxicity. A non-additive effect was also found, as the toxicity of trinitroaromatic compounds did not show higher values than the toxicity of dinitroaromatic compounds [26].

Another study was carried out by Gooch et al. [27], who reported for the first time an extended dataset of 90 NACs using the same endpoint, i.e., the 50% lethal dose concentration for rats (LD_50_). Several QSAR models were developed based on different classes of molecular descriptors including quantum chemical and topological molecular descriptors computed by DRAGON [28], PaDEL [29] and HiT-QSAR [30] software. The resulting best QSAR model was a combination of the unique indices from the different software, and gave reasonable results for the training (*R*^2^ = 0.81), internal validation (*Q*^2^ = 0.75) and test (*R*^2^ = 0.72) sets. It is also important to remark that the authors reveal some structural relationships in terms of functional groups related to toxicity. This is the case for compounds with additional hydroxyl (-OH) and methyl (CH_3_) groups showing the highest toxicity. The presence of -PO_4_ and -SO_4_ groups increases toxicity, while the presence of -NH_2_ groups can drastically reduce toxicity [27].

Later in 2020, Mondal et al. [25] used specific substructures generated by Monte Carlo method to develop QSAR models using SMILES and graph-based descriptors in a dataset of 90 NACs. This dataset is the same as previously described for the study by Gooch et al. [27]. In that work, the QSAR model shows lower values for the statistical parameters (*R*^2^*_train_* = 0.719, *Q*^2^*_train_* = 0.695; *R*^2^*_test_* = 0.739). Despite these values, the study shows interesting structural relationships to toxicity through the use of the substructures mentioned above. For example, the presence of a heteroatom with 7 out of 14 double bonded oxygens, double bonded oxygen and sp^2^ with double bond increases toxicity. On the other hand, the presence of some substructures such as *sp^3^* with branching, heteroaromatic nitrogen, and the presence of oxygen and carbon and NH_2_ groups reduces the toxicity in NACs. More details on the analysis of substructures are provided in the original literature [25].

In a similar way, Keshavarz et al. [31] used the same dataset of 90 NACs to perform a QSAR study based on constitutional descriptors such as sulphur, oxygen and molecular fragments. The best MLR model showed reasonable statistical parameters in the training (*R*^2^ = 0.858) and test (*R*^2^ = 0.857) set. The authors obtained an equation with five parameters for toxicity (−logLD_50_(*M*) = 1.599 + 0.4293*nNO_2_ − 0.4165*nS + 1.771*nP + 1.313*Tox^+^ − 2.110*Tox^−^). Three simple descriptors appear in this equation, two of which contribute positively to toxicity: *nNO_2_*, a descriptor related to the number of nitro groups, and *nP*, the number of phosphorus atoms. The descriptor *nP*, which accounts for the number of sulphur atoms, contributes negatively to toxicity. In addition, the equation contains two other adjustable parameters, *Tox^+^* and *Tox^−^*, whose interpretation in relation to the toxicity of NAC is more difficult and therefore affects the interpretation of the mechanism of the other constitutional descriptors in the equation [31]. 

More recently, Hao et al. [9] performed QSAR modeling of acute oral toxicity data in rats (LD_50_), using a dataset of 128 NACs. In this study, seven simple 2D molecular descriptors were selected for the QSAR model after applying the GA-MLR variable selection methods. They reported a squared correlation coefficient *R*^2^ of 0.748 for the training set (*n* = 101) and 0.759 for the external test set (*n* = 27). The most important descriptors were P_VSA_s_1, B06[C-F] and F09[C-N] which were positively related to toxicity, indicating that the higher values of these descriptors contributed to higher toxicity. These descriptors are related to the van der Waals surface area (P_VSA_s_1), the presence of C-F bonds at topological distance 6 (B06[C-F]), and the high frequency of C-N bonds at topological distance 9 (F09[C-N]) [9]. 

Although all of these previous papers report on the QSAR studies of the in vivo toxicity of NACs, they have certain limitations. First, they have moderate predictive power. Second, the limited size of the published datasets restricts the chemical space of the QSAR models for accurate predictions of the NACs toxicity. Finally, the influence of descriptors in non-linear models can hardly be investigated in sufficient detail. In this work, a QSAR model was developed for more than 200 NACs using acute oral toxicity of LD_50_ concentration for rats, which showed high predictive performance. The final model (ensemble model) combines the result of two Support Vector Regressions (SVR) and predicts the −logLD_50_ value of a given NAC with high accuracy. In addition, the Accumulated Local Effect (ALE) approach was used to better understand the mechanistic relationship between the descriptors involved in the models and toxicity (−log LD_50_) [32]. To the best of our knowledge, this is the first study to use ALE method to explain the mechanistic interpretation of a non-linear QSAR model.

## 2. Materials and Methods

### 2.1. Experimental Data Collection

Initially, 204 nitroaromatic compounds (NACs) with a wide range of toxicity values based on the same experimental assay were collected from the ChemIDplus [33] and PubChem [34] databases. The complete dataset can be found in the supplementary material (Table S1). All molecules were optimized by the HyperChem software [35] using the molecular mechanics method MM+. The optimization algorithm was the Polak-Ribiere (Conjugate gradient) with the termination condition RMS gradient of 0.1 kcal/(Å mol). The dataset endpoint, −logLD_50_, was calculated by converting all LD_50_ values to molar values (mol/kg) and mapping them to a negative logarithm scale. For validation purposes the dataset was split into a training set and a test set, where the training set was used for model generation. Additionally, a set of seven NACs was collected for additional external evaluation of the model performance as a true external test set. These data can be found in Table S2.

### 2.2. Generation of Descriptors 

To generate a set of descriptors, Dragon 6.0 software [28] was used. This version of Dragon provides 4885 various molecular descriptors from 0D to 3D containing 20 different molecular descriptor blocks, including topological indices, constitutional, connectivity, 3D matrix-based descriptors. Highly correlated descriptors (*R* > 0.9), constant and near constant (std < 0.1) were removed during preprocessing. All these steps were performed using Python (version 3.7.6). After eliminating correlated, constant and near constant descriptors, about 870 descriptors per NAC were used for further analysis. Because of the large differences in the scales, it can be seen that descriptors with larger range outweigh those with smaller range [12]. In this context the standard scale normalization was used as implemented in the Scikit-learn package [36] which uses the following equation (Equation (1)) to normalize the data according to their mean and standard deviation:

(1) $$x_{ij}=\frac{X_{ij}-{\bar{X}}_{j}}{\sqrt{\frac{\sum_{1}^{n}{\left(X_{ij}-{\bar{X}}_{j}\right)}^{2}}{n-1}}}$$

where *n* is the number of compounds, ${\bar{X}}_{j}$ is the mean values of the *j*^th^ descriptor, *x_ij_* and $X_{ij}$ are the normalized and original values of the *j*^th^ descriptor of the *i*^th^ compound. 

### 2.3. QSAR Modeling and Validation 

All developed QSAR models were subjected to statistical analysis evaluating the squared correlation coefficient (*R*^2^), Root Means Square Error (*RMSE*) and Mean Absolute Error (*MAE*). As a result, for each created model, the following equations were used to determine the squared correlation coefficient *R*^2^ (Equation (2)), the Root Mean Square Error (Equation (3)), the Mean Absolute Error (Equation (4)) to evaluate the goodness of fit and the Concordance Correlation Coefficient (*CCC*, Equation (6))

(2) $$R^{2}=1-\frac{\sum_{i=1}^{n}{\left(y_{i}^{obs}-y_{i}^{pred}\right)}^{2}}{\sum_{i=1}^{n}{\left(y_{i}^{obs}-{\tilde{y}}^{obs}\right)}^{2}}\;$$

(3) $$RMSE=\sqrt{\frac{\sum_{i=1}^{n}{\left(y_{i}^{obs}-y_{i}^{pred}\right)}^{2}}{n}}$$

(4) $$MAE=\frac{1}{n}\;\overset{n}{\underset{i=1}{\sum }}|y_{i}^{obs}-y_{i}^{pred}|$$

where $y_{i}^{obs}$ and $y_{i}^{pred}$ are observed and predicted values for *i*^th^ compound, accordingly, and ${\tilde{y}}^{obs}$ is the mean of observed values. We estimated the Mean Absolute Error of cross-validation *MAECV* in each example to assess model stability according to Equation (5). In Equation (6), ${\bar{y}}^{obs}$ and ${\bar{y}}^{pred}$ are the mean values for observed and predicted values.

(5) $$MAECV=\frac{1}{n}\;\overset{n}{\underset{i=1}{\sum }}|y_{i}^{obs}-y_{i}^{predcv}|$$

(6) $$CCC=\frac{2\sum_{i=1}^{n}(y_{i}^{obs}-{\bar{y}}^{obs})(y_{i}^{pred}-{\bar{y}}^{pred})}{\sum_{i=1}^{n}{\left(y_{i}^{obs}-{\bar{y}}^{obs}\right)}^{2}+\sum_{i=1}^{n}{\left(y_{i}^{pred}-{\bar{y}}^{pred}\right)}^{2}+n{\left({\bar{y}}^{obs}-{\bar{y}}^{pred}\right)}^{2}}$$

According to the OECD (Organization for Economic Co-Operation and Development) principal N0.4 for developing QSAR models “appropriate measures of goodness-of–fit, robustness and predictivity” [37], there are more criteria that must be considered to facilitate assessing a QSAR model for regulatory purposes. The model’s external predictability was evaluated with using the metrics $Q_{F1}^{2}$, $Q_{F2}^{2}$, $r_{m}^{2}$, k, k′ [38,39,40,41]. Here k, k′ are the slopes of the regression lines and should to be close to 1 [40]. The parameter $r_{m}^{2}$ is calculated from the experimental values on the ordinate axis [38], and according to Roy et al. it should be >0.5 [39,41].

We selected the best model based on the above parameters for both the training set and the test set to avoid overfitting. It is worth noting that selecting a smaller number of descriptors in the model was also considered an important parameter to reduce the complexity of the model and the computational cost. To confirm that the selected model is not close to random, the Y-scrambling test [42] was performed. In this method, the target variable is randomly shuffled to produce a dummy dataset. Therefore, there should be no correlation between the selected descriptors and the new target variable. As a result, the performance of the scrambled models should drop significantly [43]. The performance of the models is measured by their *R*^2^.

#### Support Vector Regression and Ensemble Model

**Figure 1:**
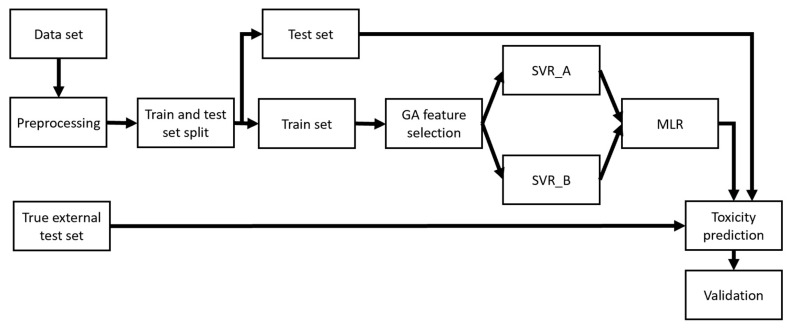
Demonstration of the methodology used to develop QSAR models.

For the construction and subsequent evaluation of QSAR models, the data were randomly divided into training and test sets in a ratio of 9:1. This ratio was chosen after experimenting with different training/test split ratios (3:1, 4:1, …, 9:1), and resulted in the best model performance and the least number of descriptors. In the preliminary phase, six structures were identified as outliers and removed from the training set. In the current study, the correlation between activity and structural descriptors was developed using the Genetic Algorithm (GA) for variable selection and Support Vector Regression (SVR) methods. As a result, GA-SVR was used for a preliminary model selection. The GA variable selection started with a population of 150 random models and 2000 iterations for evolution, with the mutation probability set to 20%. Some researchers have advocated combining learners in different methods, and their results have shown that they perform better than a single candidate learner [12,44]. In this context, after developing SVR models, two SVRs that had the best statistical parameters and robustness were used to create a hierarchical ensemble and develop a QSAR model which has substantially better performance than any single one in the hierarchy—as demonstrated previously [45]. According to the same study, a MLR model was used to refine the output of the baseline SVRs and build the ensemble model. The hierarchy of this method is shown in Figure 1. 

The SVR parameters, such as the optimization parameters C and gamma, were optimized using a grid search technique. The runtime parameters for SVR and MLR models are listed in Table 1. More information about these parameters can be found in Scikit-learn library documentation [34]

### 2.4. Analysis of Descriptors in Models

Interpreting non-linear methods/models has always been a major challenge. However, there are several techniques to make supervised machine learning models interpretable [46]. In this work, the Accumulated Local Effect (ALE) [32] was used to investigate the effect of each descriptor on the target variable. ALE is a novel alternative to the previous Partial Dependence Plot (PDP) that overcomes the problem of explaining correlated descriptors. Moreover, the ALE method is much less computationally demanding than PDP [32]. 

## 3. Results

### 3.1. Distribution of Molecular Weights and Toxicity

**Figure 2:**
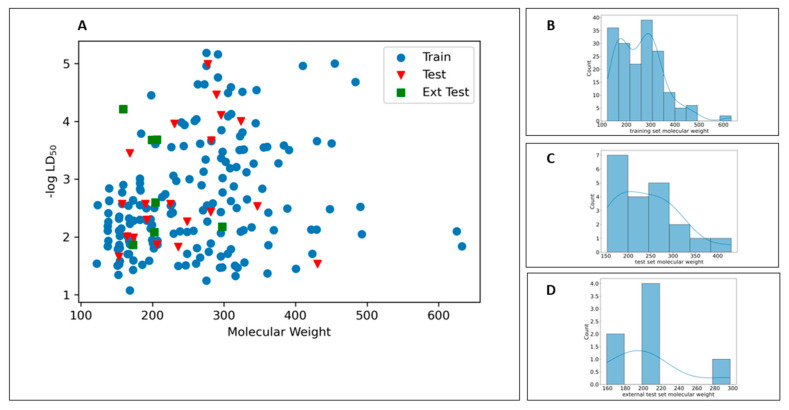
(**A**) Scatter plots of −logLD_50_ values vs. molecular weight. Histogram of molecular weights for (**B**) training set, (**C**) test set, (**D**) external test set.

The distribution of the chemical space of the dataset is crucial for predictive performance [47] of a model. In this work, the chemical space was defined using the molecular weight (MW) [9] and −logLD_50_ for all three data sets. As can be seen in Figure 2, the training data are heterogeneously distributed. It can be observed that the compounds in both the external and the true external test sets share the same chemical space as the training data. 

### 3.2. Ensemble Model

After initial pre-processing steps, 870 descriptors were extracted. Using these descriptors, several SVR-QSAR models were developed and then two SVR models with the best statistical parameters were selected. These two SVR models are named SVR_A and SVR_B and have 11 and 8 descriptors, respectively. The ensemble was created by applying MLR to the results obtained from the SVR models to refine the prediction. The statistical parameters for the selected models and ensemble are presented in Table 2. Statistically, our ensemble model performed better than each individual model on the training, test and external test sets, as shown by the parameters in Table 2.

**Figure 3:**
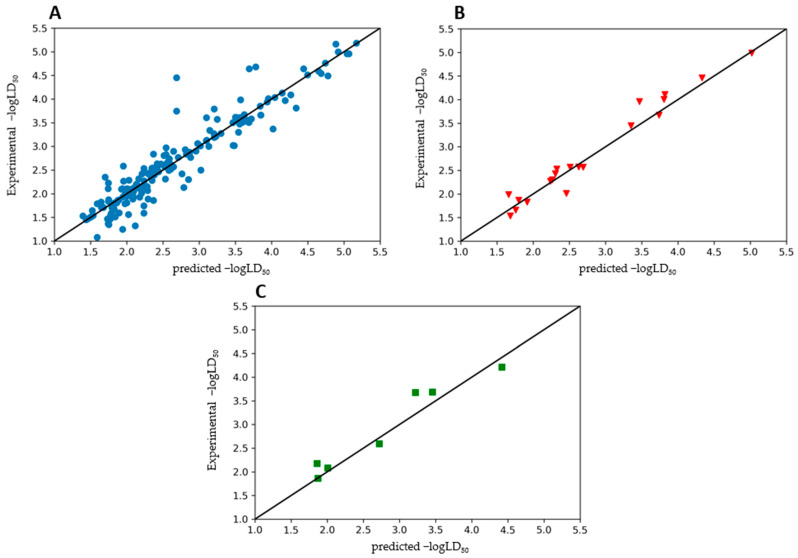
Experimental versus predicted −logLD_50_ values obtained by the ensemble model for the training (**A**), test (**B**) and external test (**C**) sets.

SVR_A and SVR_B are performed approximately the same for the training set, but at the same time, SVR_A has a better performance for the test set with *R*^2^ = 0.92. This can be seen from the fact that the residual errors are smaller than those of SVR_B. In contrast, SVR_B showed better performance on the external test set. When the ensemble model was applied to the external test set, better performance results were obtained, indicating that this model has high predictive power and is well trained. Figure 3 shows the predicted versus experimental −logLD_50_ for the training set (Figure 3A), the test set (Figure 3B) and the true external validation set (Figure 3C). In each scatter plot, the black solid line shows the associated regression line to the data points that confirm these performance results for the ensemble model.

A similar improvement in performance was obtained by applying the ensemble model in another work, where a QSAR modelling of intrinsic solubility of chemicals was studied, which was published in a recent paper by Lovrić et al. [48]. An RMSE (test) of 0.67 log units and an *R*^2^ (test) of 0.81 (*n* = 166) were obtained by the ensemble model constructed as a simple average of the predictions of the two best ML models. These individual models yielded RMSE (test) values of 0.70 and 0.72, i.e., *R*^2^ (test) values of 0.80 and 0.78, respectively. The quality of the models in mentioned study is expressed by the parameters that measure an agreement (*R*^2^), but also by the parameters that estimate the standard error of the estimate or prediction (*RMSE* and *MAE*) as the basic model validation measure. It worth noting that for all models and for all sets (training set, test set and external test set), higher *R*^2^ values were always associated with lower *RMSE* values, indicating their consistency and stability. This is a desirable predictive property of the model, especially for external data sets, as Lučić et al. have shown with examples (in Table 2 [49]) that with very small changes in external dataset it is possible for the *R*^2^ to increase even in situations where *RMSE* decreases—in a case where an extremely bad prediction with error being greater than 2*RMSE was obtained in one additional example.

**Figure 4:**
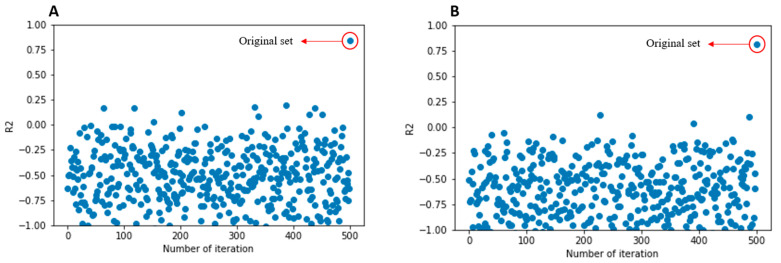
*Y*-scrambling plots for SVR_A and SVR_B models.

In current study the model showed a very good performance and validation values. For example, Figure 4 shows the scatter plot of the *y*-scrambling diagram of the two basic SVR models with 500 iterations. It can be seen that the original model is very robust since all random data sets do not yield acceptable *R*^2^, confirming that the model is not the result of chance correlation.

The descriptors that were selected by the GA technique and used in each SVR model can be found in Table 3.

As can be seen in Table 3, SVR_A and SVR_B have three descriptors in common. The first is AVS_B(e), the average vertex sum from the Burden matrix weighted by Sanderson’s electronegativity, implying that electronegativity may play a crucial role in toxicity. The second common descriptor for the two models is the leverage-weighted autocorrelation of lag 7/weighted by I-state (HATS7s), another molecular descriptor related to electronic effects, and the third is Eta_sh_y (Eta and shape index), a shape-related descriptor. 

**Figure 5:**
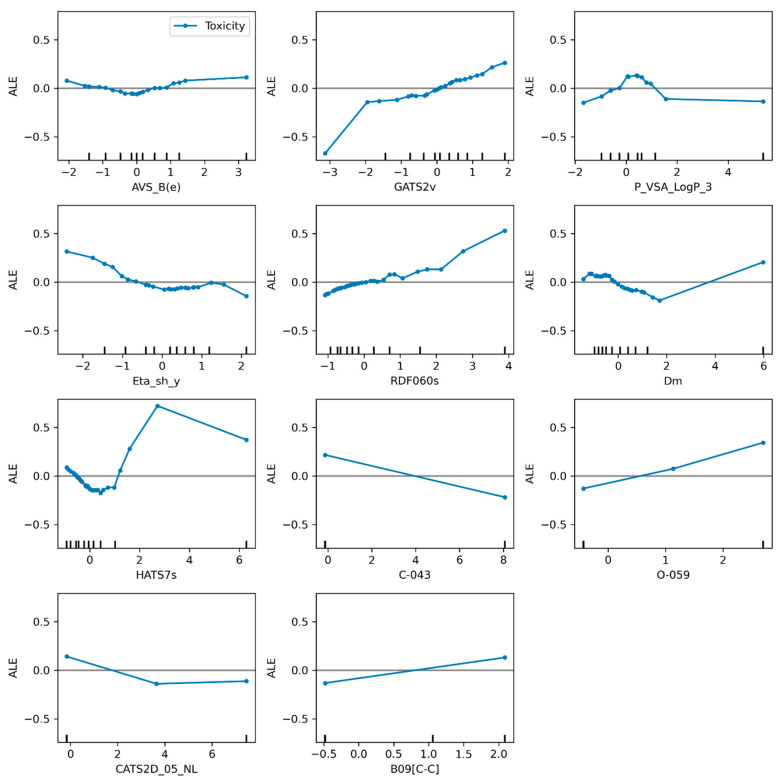
Descriptors weights represented by ALE plot for the SVR_A model.

**Figure 6:**
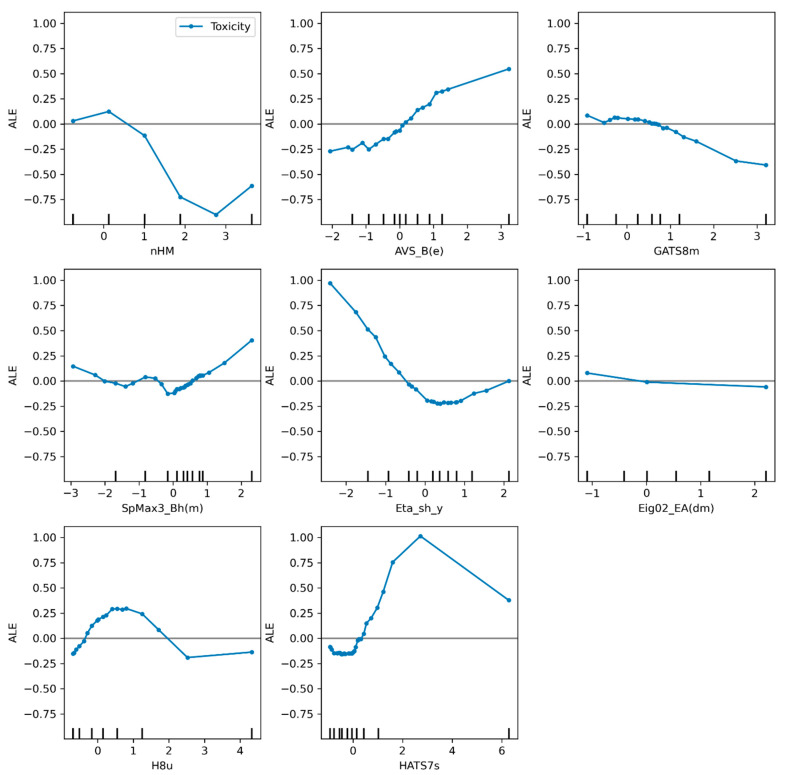
Descriptors weights represented by ALE plot for the SVR_B model.

From these molecular descriptors, which are unique to each model, it can be seen that SVR_A has more volume- and shape-related descriptors such as GATS2v, a molecular descriptor weighted by van der Waals volume, and RDF060s which uses a radial distribution function. In the case of SVR_B, there are three descriptors related to the mass of the molecules: GAS8m (Geary autocorrelation of lag 8 weighted by mass), the nHM descriptor that considers the heavy atoms, and SpMax3_Bh(m), another molecular descriptor with a matrix weighted by mass. It should be emphasized that although both SVR_A and SVR_B include mass-, volume-, and electronic-related molecular descriptors as main features for describing toxicity, a topological descriptor such as B09[C-C] (presence/absence of C-C at topological distance 9) helps to describe the influence of large chains on the toxicity of molecules. Figure 5 and Figure 6 show the result of the method ALE for the models SVR_A and SVR_B.

As can be seen in the case of the SVR_A model (Figure 5), the descriptors AVS_B(e), CATS2D_05_NL and B09[C-C] have no remarkable influence on toxicity. With the increase of smaller values of the P_VSA_LogP_3 descriptor, the toxicity values increase, but for descriptor values above 0.5, the average predictions decrease, and for values above 1.5, the descriptor does not have much influence on the predictions. The following three descriptors GATS2v, RDF060s and O-059 show a strong positive effect on toxicity meaning that increasing value of these descriptors will increase the toxicity. In the case of GATS2v and RDF060s descriptors these effects are related to surface distributions of positive charges, negative charges, H-bond donors, H-bond acceptors, and regions of high polarizability, which indirectly increase the lipophilicity and hence the toxicity. The molecular descriptor O-059 is related to the nitro-group properties by contributing to reduce the electron density of the aromatic rings, which means that the nitro-compounds with substructures containing oxygens show strong electron-withdrawing effect [50]. These make nitroaromatic compounds more capable to attack nucleophiles at aromatic ring carbons, and hence increasing the toxicity [51].

An interesting case is descriptor Dm where toxicity values decrease with increasing values of up to Dm = 2, where toxicity starts to increase with higher values of Dm. 

HATS7s is a unique case because it shows 3 different zones in the ALE plot. The first trend shows a decrease in toxicity with increasing values of the descriptor (HATS7s), in the second trend line of the graph a strong effect is observed with a high increase in toxicity values with the increase in descriptor values and in the last trend, when the HATS7s value is above 3, the higher the descriptor values the lower the prediction. This is in accordance with electronic effects that increase toxicity by increasing the ability of nitrocompounds to act as electrophilic agents. 

For the two remaining descriptors in the SVR_A model, Eta_sh_y and C-043, a strong negative effect on toxicity is observed when their values were increased.

The ALE plot of the SVR_B model also shows interesting results that provide some clues for interpreting the factors that influence toxicity. Prior to this method, factors were analyzed only by considering the values of regression coefficients [52,53,54,55]. As described above for the previous model, there are some descriptors that have no influence on toxicity. In the case of the SVR_B model, Eig02_EA(dm) has no significant influence on the toxicity predictions. The descriptors Eta_sh_y and nHM have a negative influence on toxicity. However, this trend is interrupted by a slight increase in toxicity values for the highest values of these descriptors. The descriptor Eta_sh_y is related to the van der Waals surface area which is indirectly related to lipophilicity—the higher this factor, the greater the toxicity [9]. The nHM descriptor denotes the number of heavy atoms with principal quantum number *L* larger than 2, which corresponds to a molecular size that is indirectly associated with lipophilicity and increase in lipophilicity may lead to increase in the toxicity of the nitrocompounds. 

Increasing the value of the descriptor SpMax3_Bh(m) has no significant effect on toxicity until it reaches value above zero, where it begins to show a positive correlation with toxicity. This Burden descriptor [56] is related to surface distribution of positive charges, negative charges, H-bond donors, H-bond acceptors, and regions of high polarizability, which indirectly increase the lipophilicity and thus toxicity. A completely different behavior is shown by the H8u descriptor, where an increase in toxicity is observed at the lowest values of the descriptor, but then starts to decrease from 0.5 to about 2.5. for the values above 2.5 there is no significant effect on toxicity. The lower values of the descriptor GATS8m do not affect toxicity, but for the descriptor values above 1, the toxicity values decrease showing a negative correlation with the higher values of the descriptor. Finally, there are two descriptors common to both the SVR_A and SVR_B models where notable patterns can be observed. First, as already discussed for the SVR_A model, the descriptor AVS_B(e) shows no effect on toxicity. However, for the SVR_B model, the same descriptor shows a strong positive effect on toxicity, i.e., the higher the descriptor values, the higher the toxicity in line with the same expected effect of SpMax3_Bh(m) descriptor, the other Burden descriptor included in the SVR_B model. The second common descriptor for both SVR models, HATS7s, shows the same pattern for both models with three different zones: decrease in toxicity in the first zone, increase in toxicity in the second zone and decrease in toxicity values in the third zone with the higher values of the descriptor. These relationship-related interpretations of the ALE plots could provide evidence for the development of less toxic compounds based on ALE plot intervals of the descriptors where lower toxicity is observed. 

## 4. Conclusions

In this work, an ensemble QSAR model comprising two SVRs models is developed that predicts the in vivo toxicity of nitroaromatic compounds. The models were tested by a number of testing methods [38] and all statistical parameters of this model show that the model is robust and accurate, with *R*^2^ = 0.88 for the training set and *R*^2^ = 0.95 for the test set. Additionally, the contribution of each descriptor to toxicity was discussed using the Accumulated Local Effect (ALE) approach. This novel approach worked very well in this study as it was able to show the intervals of the linear relationship between the descriptors and toxicity for non-linear models such as Support Vector Regression. The developed ensemble QSAR model has eight descriptors showing strong positive effects on toxicity, while five descriptors show negligible effects, and three descriptors show a negative effects. It is important to emphasize that HATS7s is a common descriptor for SVR_A and SVR_B. The ALE plot of both models shows the same pattern for this descriptor. The obtained results describe the structural relationship between toxicity and molecular descriptors in developed non-linear models that could be helpful in assessment of the toxicity of existing nitroaromatic compounds and development of less toxic analogues. Moreover, the applied ALE approach might provide some mechanistic explanations to better describe the effects of the molecular descriptors in supervised black-box machine learning models.

## Figures and Tables

**Table 1:** Runtime parameters for SVR and MLR models.

Method/Model	Runtime Parameters
SVR_A and SVR_B	Kernel = ’rbf’, degree = 3, gamma = ’auto’, coef 0 = 0.0, tol = 0.001, C = 5.0, epsilon = 0.1, shrinking = Ture, cache_size = 200, verbose = False, max_iter = −1
MLR	Fir_intercept = True, normalize = ’False’, copy_X = True, n_jobs = −1, positive = False

**Table 2:** Statistical parameters of SVR_A and SVR_B models.

Parameters	Regression Model		Ensemble Model
	SVR_A	SVR_B	
No. of descriptors	11	8	_
*R*^2^ (training)	0.83	0.81	0.88
*RMSE* (training)	0.111	0.127	0.093
*MAE* (training)	0.221	0.226	0.199
*MAECV*(5-Fold)	0.484	0.486	0.480
*R*^2^ (test)	0.92	0.85	0.95
*RMSE* (test)	0.056	0.096	0.041
*MAE* (test)	0.191	0.250	0.155
*CCC* (test)	0.968	0.946	0.978
*R*^2^ (external test)	0.74	0.88	0.92
*RMSE* (external test)	0.132	0.123	0.061
*MAE* (external test)	0.320	0.319	0.202
*CCC* (external test)	0.898	0.931	0.961
$Q_{F1}^{2}$	0.945	0.906	0.960
$Q_{F2}^{2}$	0.943	0.903	0.958
$r_{m}^{2}$	0.510	0.536	0.560
k	0.955	0.981	0.975
k′	1.041	1.007	1.021

**Table 3:** Descriptors involved in each SVR model and the corresponding definition.

Descriptor	SVR_A	SVR_B	Definition and Scope	Descriptor Type
AVS_B(e)	X	X	average vertex sum from Burden matrix weighted by Sanderson electronegativity	2D matrix-based descriptors
HATS7s	X	X	leverage-weighted autocorrelation of lag 7/weighted by I-state	GETAWAY descriptors
Eta_sh_y	X	X	Eta y shape index	ETA indices
GATS2v	X		Geary autocorrelation of lag 2 weighted by van der Waals volume	2D autocorrelations
GATS8m		X	Geary autocorrelation of lag 8 weighted by mass	2D autocorrelations
P_VSA_LogP_3	X		P_VSA-like on LogP, bin 3	P_VSA-like descriptors
nHM		X	number of heavy atoms	Constitutional indices
RDF060s	X		Radial Distribution Function—060/weighted by I-state	RDF descriptors
Dm	X		D total accessibility index/weighted by mass	WHIM descriptors
H8u		X	H autocorrelation of lag 8/unweighted	GETAWAY descriptors
O-059	X		Al-O-Al	Atom-centred fragments
B09[C-C]	X		Presence/absence of C—C at topological distance 9	2D Atom Pairs
SpMax3_Bh(m)		X	largest eigenvalue *n*. 3 of Burden matrix weighted by mass	Burden eigenvalues
CATS2D_05_NL	X		CATS2D Negative-Lipophilic at lag 05	CATS 2D
Eig02_EA(dm)		X	eigenvalue *n*. 2 from edge adjacency mat. weighted by dipole moment	Edge adjacency indices
C-043	X		X--CR.X	Atom-centred fragments

## Data Availability

Not applicable.

## Supplementary Materials

The following supporting information can be downloaded at: https://www.mdpi.com/article/10.3390/toxics10120746/s1, Table S1: Data set collected for further use in developing the ensemble QSAR model; Table S2: True external test set for evaluating the ensemble QSAR model.
