# Supplementary material for: In Silico Prediction of the Toxicity of Nitroaromatic Compounds: Application of Ensemble Learning QSAR Approach
Source: Toxics. 2022 Dec 1;10(12):746. doi: 10.3390/toxics10120746 (PMC9786026; doi:10.3390/toxics10120746)
Supplement: Supplementary file 1 [file toxics-10-00746-s001.zip › toxics-1998766-supplementary.pdf]

In Silico Prediction of the Toxicity of Nitroaromatic Compounds: Application of Ensemble Learning QSAR Approach

Amirreza Daghighi [1,2], Gerardo M. Casanola-Martin [2], Troy Timmerman [2,3], Dejan Milenković [4], Bono Lucic [5]*, Bakhtiyor Rasulev [1,2]*

[1] Biomedical Engineering Program, North Dakota State University, Fargo, ND 58105, USA

[2] Department of Coatings and Polymeric Materials, North Dakota State University, Fargo, ND 58102, USA

[3] Department of Computer Science, North Dakota State University, Fargo, ND 58105, USA

[4] Department of Science, Institute for Information Technologies, University of Kragujevac, Kragujevac, Serbia

[5] NMR Centre, Rudjer Boskovic Institute, Zagreb, Croatia

Table S1. Data set for developing the ensemble QSAR model.

ID	SMILES	CAS-ID	-logLD50 (rat toxicity)	Ensemble prediction	SVR_A prediction	SVR_B prediction	Set
1	[O-][N+](=O)C1=CC=CC=C1C2=CC=CC=C2	86-00-0	2.21	2.14	2.11	2.31	Train
2	[O-][N+](=O)C1=CC(=C(F)C=C1F)[N+]([O-])=O	327-92-4	3.61	3.57	3.51	3.49	Train
3	NC1=C(Cl)C=C(C=C1)[N+]([O-])=O	121-87-9	1.43	1.75	1.73	1.98	Train
4	[O-][N+](=O)C1=CC(=C(Cl)C=C1)[N+]([O-])=O	97-00-7	2.50	3.02	3.11	2.85	Train
5	CN1C(=O)C2=C(C=C(C=C2)[N+]([O-])=O)C1=O	41663-84-7	1.87	1.80	1.99	1.72	Test
6	[O-][N+](=O)C1=CC=C(F)C=C1F	446-35-5	2.90	2.96	3.10	2.71	Train
7	OC(=O)C1=C(C2=C(C=C1)C(=O)C3=CC=CC=C3C2=O)[N+]([O-])=O	128-67-6	2.17	2.23	2.27	2.27	Train
8	[O-][N+](=O)C1=CC2=C(C=C1)C3=C(C=C(C=C3C2=O)[N+]([O-])=O)[N+]([O-])=O	129-79-3	1.50	1.49	1.60	1.60	Train
9	[O-][N+](=O)C1=CC(=CC=C1Cl)Cl	89-61-2	2.28	2.35	2.38	2.38	Train

10	NC1=C(O)C=CC(=C1)[N+]([O-])=O	99-57-0	1.81	1.86	1.91	1.98	Train
11	NC1=C(O)C=C(C=C1)[N+]([O-])=O	97-52-9	1.59	1.78	1.80	1.97	Train
12	CC1=CC(=C(O)C=C1)[N+]([O-])=O	119-33-5	1.66	1.76	1.79	1.92	Test
13	NC1=CC=CC=C1[N+]([O-])=O	88-74-4	1.94	2.09	2.04	2.31	Train
14	COC1=C(C=CC=C1)[N+]([O-])=O	91-23-6	2.32	2.17	2.22	2.21	Train
15	[O-][N+](=O)C1=CC=C2C=CC=CC2=C1	581-89-5	1.60	1.89	2.15	1.70	Train
16	NC1=CC(=C(N)C=C1)[N+]([O-])=O	5307-14-2	1.86	1.88	1.96	1.94	Train
17	NC1=CC(=CC=C1)[N+]([O-])=O	99-09-2	2.41	2.31	2.37	2.31	Train
18	CC1=CC(=CC=C1)[N+]([O-])=O	99-08-1	2.11	2.00	2.01	2.16	Train
19	O[S](=O)(=O)C1=C(/C=C/C2=C(C=C(C=C2)[N+]([O-])=O)[S](O)(=O)=O)C=CC(=C1)[N+]([O-])=O	128-42-7	1.53	1.69	1.85	1.67	Test
20	NC1=CC(=C(O)C=C1)[N+]([O-])=O	119-34-6	2.02	2.05	2.11	2.11	Train
21	CC1=C(C=C(N)C=C1)[N+]([O-])=O	119-32-4	1.35	1.75	1.87	1.78	Train
22	NC1=C(C=C(Cl)C=C1)[N+]([O-])=O	89-63-4	2.64	2.42	2.48	2.41	Train
23	OC(=O)C1=CC=C(Cl)C(=C1)[N+]([O-])=O	96-99-1	1.81	1.83	1.91	1.91	Train
24	[O-][N+](=O)C1=C(Cl)C=CC(=C1)C(F)(F)F	121-17-5	2.40	2.38	2.41	2.42	Train
25	NC1=CC=C(C=C1)[N+]([O-])=O	100-01-6	2.27	1.95	1.87	2.25	Train
26	[O-][N+](=O)C1=CC=C(C=C1)C(Cl)=O	122-04-3	1.52	1.51	1.62	1.62	Train
27	COC1=C(N)C=C(C=C1)[N+]([O-])=O	99-59-2	1.87	1.75	1.88	1.77	Train
28	CC(=O)CC(C1=CC=C(C=C1)[N+]([O-])=O)C2=C(O)C3=CC=CC=C3OC2=O	152-72-7	2.84	2.82	2.74	2.94	Train
29	OC(=O)C1=C(C=CC(=C1)OC2=CC=C(C=C2Cl)C(F)(F)F)[N+]([O-])=O	50594-66-6	2.42	2.21	2.31	2.19	Train
30	CC[P](=S)(OC(C)C)OC1=CC=C(C=C1Cl)[N+]([O-])=O	328-04-1	4.01	3.81	3.84	3.54	Test
31	COC1=CC=CC2=C3C4=C(OCO4)C=C(C(O)=O)C3=C(C=C12)[N](=O)=O	313-67-7	3.27	3.20	3.17	3.17	Train
32	C[N]1C(=NC=C1[N+]([O-])=O)\C=C\C2=NC(=NC=C2)N	62973-76-6	1.51	1.84	1.61	2.36	Train

33	COC1=CC(=CC=C1N)[N](=O)=O	97-52-9	2.23	1.75	2.13	1.41	Train
34	[O-][N+](=O)C1=CC=C(CCl)C=C1	100-14-1	1.98	1.97	2.07	1.99	Train
35	[O-][N+](=O)C1=CC=C(C=C1)N=C=O	100-28-7	2.01	2.46	2.34	2.70	Test
36	OC(=O)C1=CC(=CC(=C1Cl)[N+]([O-])=O)Cl	88-86-8	1.83	1.92	1.99	1.99	Test
37	COC1=CC(=C(NC(C)=O)C=C1N(CCOC(C)=O)CCOC(C)=O)N=NC2=C(Br)C=C(C=C2[N+]([O-])=O)[N+]([O-])=O	3618-72-2	2.10	1.93	2.00	2.00	Train
38	OCC(NC(=O)C(Cl)Cl)C(O)C1=CC=C(C=C1)[N+]([O-])=O	56-75-7	2.09	2.06	2.06	2.19	Train
39	[O-][N+](=O)C1=CC2=C(NC(=O)CN=C2C3=CC=CC=C3Cl)C=C1	1622-61-3	1.32	2.12	1.77	2.75	Train
40	OC1=C(C=C(C=C1C2CCCCC2)[N+]([O-])=O)[N+]([O-])=O	131-89-5	3.61	3.58	3.51	3.51	Train
41	CC1=C(C=C(C=C1C(N)=O)[N+]([O-])=O)[N+]([O-])=O	148-01-6	2.57	2.63	2.58	2.73	Test
42	CCC(C)C1=CC(=CC(=C1O)[N+]([O-])=O)[N+]([O-])=O	88-85-7	3.98	3.57	3.61	3.36	Train
43	CC1=C(O)C(=CC(=C1)[N+]([O-])=O)[N+]([O-])=O	534-52-1	4.45	2.69	2.62	2.80	Train
45	CN1C(=O)CN=C(C2=CC=CC=C2F)C3=C1C=CC(=C3)[N+]([O-])=O	1622-62-4	2.88	2.87	2.78	2.98	Train
46	CO[P](=S)(OC)OC1=CC(=C(C=C1)[N+]([O-])=O)C(F)(F)F	363-97-3	3.12	3.10	3.10	3.02	Train
47	[O-][N+](=O)C1=CC(=CC=C1)C(F)(F)F	98-46-4	2.50	2.58	2.60	2.60	Train
48	CC(=O)C1=CC=CC(=C1)[N+]([O-])=O	121-89-1	1.71	1.79	1.93	1.81	Train
49	CC1=C(C2=C(C=C1[N](=O)=O)C(C)(C)CC2(C)C)[N](=O)=O	116-66-5	1.75	2.24	2.24	2.34	Train
50	COC1=C(C=C(C(=C1[N+]([O-])=O)C)[N+]([O-])=O)C(C)(C)C	83-66-9	2.90	2.65	2.56	2.80	Train
51	CC(=O)C1=C(C)C(=C(C(=C1C)[N+]([O-])=O)C(C)(C)C)[N+]([O-])=O	81-14-1	1.47	1.73	1.65	2.06	Train
52	CC1=C(C)C(=C(C(=C1C)[N](=O)=O)C(C)(C)C)[N](=O)=O	145-39-1	1.65	1.52	1.55	1.75	Train

53	CC1=C(C(=C(C(=C1[N+]([O-])=O)C(C)(C)C)[N+]([O-])=O)C)[N+]([O-])=O	81-15-2	1.47	1.46	1.57	1.57	Train
55	OC1=CC=C(Cl)C=C1C(=O)NC2=CC=C(C=C2Cl)[N+]([O-])=O	50-65-7	2.12	2.09	2.22	2.05	Train
56	COC(=O)C1=C(C)NC(=C(C1C2=CC=CC=C2[N+]([O-])=O)C(=O)OC)C	21829-25-4	2.53	2.33	2.42	2.28	Test
57	[O-][N+](=O)C1=CC=C2NC(=O)CN=C(C3=CC=CC=C3)C2=C1	146-22-5	2.53	2.24	2.23	2.36	Train
58	[O-][N+](=O)C1=CC=C(OC2=C(Cl)C=C(Cl)C=C2)C=C1	1836-75-5	2.58	1.95	2.14	1.85	Train
59	OC1=CC=C(C=C1Cl)[N+]([O-])=O	619-08-9	2.29	2.03	2.18	1.96	Train
60	CC(C)OC(=O)C1=CC(=CC(=C1)[N](=O)=O)C(=O)OC(C)C	10552-74-6	1.66	1.83	2.14	1.56	Train
61	OC1=C2N=CC=CC2=C(C=C1)[N+]([O-])=O	4008-48-4	2.57	2.69	2.44	3.07	Test
63	NC1=C(N)C=C(C=C1)[N+]([O-])=O	99-56-9	2.35	1.70	1.82	1.76	Train
64	CC1=C(C=C(C=C1)[N+]([O-])=O)[S](Cl)(=O)=O	121-02-8	1.50	1.78	1.61	2.23	Train
65	CCO[P](=O)(OCC)OC1=CC=C(C=C1)[N+]([O-])=O	311-45-5	5.18	5.17	4.75	5.28	Train
66	CO[P](=S)(OC)OC1=CC=C(C=C1)[N+]([O-])=O	298-00-0	4.64	4.44	4.16	4.51	Train
67	CC(C)C1=C(O)C(=CC(=C1)[N+]([O-])=O)[N+]([O-])=O	118-95-6	3.56	3.65	3.66	3.46	Train
68	OC1=CC=C(NC2=CC=C(C=C2[N+]([O-])=O)[N+]([O-])=O)C=C1	119-15-3	1.25	1.94	2.23	1.69	Train
69	ClCCO[P](=O)(OCCCl)OC1=CC=C(C=C1)[N](=O)=O	311-44-4	3.97	4.19	4.07	4.07	Train
70	OC1=C(C=C(C=C1[N+]([O-])=O)[N+]([O-])=O)[N+]([O-])=O	88-89-1	3.06	2.98	2.96	2.96	Train
71	[O-][N+](=O)C1=CC=C(C=C1)C2=CC=CC=C2	92-93-3	1.95	2.00	2.08	2.05	Train

72	[O-][N+](=O)C1=CC=C(NC(=O)NCC2=CC=CN=C2)C=C1	53558-25-1	4.64	3.69	4.04	2.99	Train
73	O=[N](=O)C1=CC=C(OCC2CO2)C=C1	5255-75-4	1.59	2.24	2.33	2.21	Train
74	NC1=C(C=C(C=C1)[N+]([O-])=O)[N+]([O-])=O	97-02-9	2.81	2.51	2.35	2.81	Train
75	[O-][N+](=O)C1=CC(=CC(=C1Cl)[N+]([O-])=O)C(F)(F)F	393-75-9	2.46	2.44	2.49	2.44	Train
76	COC1=CC=C(C=C1)[N+]([O-])=O	100-17-4	1.82	1.65	1.75	1.72	Train
77	[O-][N+](=O)C1=CC=C(C=O)C=C1	555-16-8	1.51	1.50	1.61	1.61	Train
78	CCOC1=CC=C(C=C1)[N+]([O-])=O	100-29-8	1.71	1.80	1.94	1.81	Train
79	OC1=CC=C(C=C1C=O)[N+]([O-])=O	97-51-8	2.32	2.33	2.48	2.22	Train
80	COC1=CC=C(N)C(=C1)[N+]([O-])=O	96-96-8	1.08	1.59	1.86	1.44	Train
81	NC1=CC=C(/C=C/C2=CC=C(C=C2[S](O)(=O)=O)[N+]([O-])=O)C(=C1)[S](O)(=O)=O	119-72-2	1.45	1.44	1.55	1.55	Train
82	CCC(C)C1=CC(=CC(=C1OC(=O)C=C(C)C)[N+]([O-])=O)[N+]([O-])=O	485-31-4	3.75	2.69	2.55	2.90	Train
83	CO[P](=S)(OC)OC1=CC=C(C(=C1)Cl)[N+]([O-])=O	500-28-7	3.02	3.48	3.49	3.33	Train
84	NC1=C(Cl)C=C(C=C1Cl)[N+]([O-])=O	99-30-9	1.94	1.94	2.00	2.04	Train
85	CC(=O)C1=CC=CC=C1[N+]([O-])=O	577-59-3	2.01	2.20	2.34	2.11	Train
86	CO[P](=O)(OC)OC1=CC(=C(C=C1)[N](=O)=O)C	2255-17-6	4.04	4.04	3.94	3.94	Train
87	CC(C)C(=O)NC1=CC(=C(C=C1)[N+]([O-])=O)C(F)(F)F	13311-84-7	2.55	2.36	2.54	2.18	Train
88	C[S](=O)(=O)NC1=C(OC2=CC=CC=C2)C=C(C=C1)[N](=O)=O	51803-78-2	3.19	3.21	3.09	3.29	Train
90	[O-][N+](=O)C1=C(C=CC=C1Cl)C2=C[NH]C=C2Cl	1018-71-9	2.11	1.94	2.01	2.01	Train
91	NC1=CC=C(C=C1)C2=CC=C(N)C=C2[N+]([O-])=O	2243-78-9	2.06	2.01	2.16	1.96	Train
92	NC1=CC=C(Cl)C(=C1)[N+]([O-])=O	635-22-3	2.63	2.47	2.52	2.46	Train

93	NC1=C(C=C(Cl)C=C1[N+]([O-])=O)[N+]([O-])=O	5388-62-5	2.74	2.59	2.58	2.64	Train
94	OC1=C(C=CC=C1)[N+]([O-])=O	88-75-5	2.62	2.43	2.42	2.52	Train
95	[O-][N+](=O)C1=CC=CC(=C1)[N+]([O-])=O	99-65-0	3.45	3.35	3.31	3.29	Test
96	CC1=C(C=C(C=C1N)[N+]([O-])=O)[N+]([O-])=O	35572-78-2	2.15	2.28	2.21	2.48	Train
97	CC1=C(C=C(N)C=C1[N+]([O-])=O)[N+]([O-])=O	19406-51-0	2.31	2.53	2.41	2.76	Train
98	CC1=C(C=C(C=C1)[N+]([O-])=O)[N+]([O-])=O	121-14-2	2.83	2.54	2.53	2.61	Train
99	NC1=CC(=C(C=C1Cl)[N+]([O-])=O)Cl	6627-34-5	1.87	1.89	1.97	1.96	Train
100	NC1=NC(=C(C=C1)[N+]([O-])=O)Cl	84487-03-6	1.99	1.66	1.64	1.92	Test
101	[O-][N+](=O)C1=CC=CC(=C1)Cl	121-73-3	2.57	2.64	2.64	2.67	Train
102	NC1=CC=C(C(=C1)N)[N+]([O-])=O	5131-58-8	1.53	1.39	1.43	1.63	Train
103	OC(=O)C1=CC(=CC(=C1O)[N](=O)=O)[N](=O)=O	609-99-4	2.42	2.76	2.80	2.71	Train
104	[O-][N+](=O)C1=CN=C(Cl)C(=C1)[N+]([O-])=O	2578-45-2	3.61	3.10	3.13	2.99	Train
105	OC1=C(C=C(C=C1)[N+]([O-])=O)[N+]([O-])=O	51-28-5	3.79	3.21	3.29	2.99	Train
106	CC1=CC=CC(=C1[N+]([O-])=O)[N+]([O-])=O	602-01-7	2.30	2.85	2.92	2.73	Train
108	CC1=C(C=CC=C1[N+]([O-])=O)[N+]([O-])=O	606-20-2	3.01	3.02	2.96	3.04	Train
109	CC1=CC(=CC(=C1)[N+]([O-])=O)[N+]([O-])=O	618-85-9	2.93	2.81	2.79	2.83	Train
110	CC1=C(C=C(C=C1[N+]([O-])=O)[N+]([O-])=O)[N+]([O-])=O	118-96-7	2.57	2.52	2.52	2.58	Train
111	CCCCN1C(=O)C2=CC=C(C=C2C1=O)[N](=O)=O	54395-37-8	2.27	2.24	2.16	2.46	Test
112	NC(=O)C1=CC=C(C=C1)[N+]([O-])=O	619-80-7	2.54	2.34	2.34	2.44	Train
113	CCCCN1C(=O)C2=C(C1=O)C(=CC=C2)[N+]([O-])=O	54395-36-7	2.09	2.03	2.03	2.19	Train

114	NC(=O)C1=CC=C(Cl)C(=C1)[N+]([O-])=O	16588-06-0	1.89	2.08	2.30	1.91	Train
115	CC1=CC(=CC=C1[N+]([O-])=O)[N+]([O-])=O	619-15-8	2.55	2.42	2.45	2.45	Train
116	[O-][N+](=O)C1=CC=C(Cl)C=C1	100-00-5	2.57	2.51	2.48	2.61	Test
117	NC1=CC=C(F)C(=C1)[N+]([O-])=O	364-76-1	2.15	2.20	2.25	2.26	Train
118	OC1=CC=C(C=C1)[N+]([O-])=O	100-02-7	2.84	2.37	2.36	2.46	Train
119	NC1=C(C=C(C=C1Br)[N+]([O-])=O)[N+]([O-])=O	1817-73-8	1.81	2.00	1.91	2.29	Train
120	[O-][N+](=O)C1=C(Cl)C(=C(Cl)C(=C1Cl)Cl)Cl	82-68-8	2.43	2.29	2.33	2.33	Train
121	[O-][N+](=O)C1=C(Cl)C=CC=C1	88-73-3	2.77	2.71	2.67	2.78	Train
122	[O-][N+](=O)C1=C(Cl)C(=CC(=C1Cl)Cl)Cl	117-18-0	1.54	1.77	1.87	1.84	Train
123	[O-][N+](=O)C1=CC=C(Cl)C(=C1)Cl	99-54-7	2.30	2.26	2.29	2.33	Test
124	OC1=CC=CC(=C1)[N+]([O-])=O	554-84-7	2.63	2.60	2.67	2.53	Train
125	[O-][N+](=O)C1=CC=CC=C1	98-95-3	2.55	2.57	2.58	2.60	Train
126	CC1=CC(=CC=C1[N+]([O-])=O)O	2581-34-2	2.11	1.74	1.87	1.77	Train
127	CC1=CC=CC=C1[N+]([O-])=O	88-72-2	2.19	2.13	2.19	2.18	Train
128	CC1=CC(=C(C)C=C1)[N+]([O-])=O	89-58-7	1.79	1.59	1.69	1.69	Train
129	NC(=O)C1=CC=CN=C1	98-92-0	1.54	1.54	1.64	1.64	Train
130	[O-][N+](=O)C1=CC2=C3C=CC=CC3=CC=C2O1	69267-51-2	2.69	2.57	2.59	2.59	Train
131	CC1=CC=C(C=C1)[N+]([O-])=O	99-99-0	1.84	1.74	1.76	1.94	Train
132	CCN(CC1=C(Cl)C=CC=C1F)C2=C(C=C(C=C2[N+]([O-])=O)C(F)(F)F)[N+]([O-])=O	62924-70-3	2.13	2.25	2.23	2.40	Train
133	CCOC(=O)COC(=O)C1=C(C=CC(=C1)OC2=C(Cl)C=C(C=C2)C(F)(F)F)[N+]([O-])=O	77501-90-7	2.48	2.34	2.38	2.38	Train
134	OC1=C(NN=C2C(=O)C3=C(C=C(C=C3NC4=NC(=NC(=N4)Cl)Cl)[S](O)(=O)=O)C	73826-58-1	1.84	1.86	1.94	1.94	Train

	=C2[S](O)(=O)=O)C=C(C=C1)[N+]([O-])=O						
135	OC(=O)C1=C(Cl)C=CC(=C1Cl)Cl	50-31-7	2.54	2.41	2.44	2.44	Train
136	OC(=O)C1=CC=C(C=C1)[N+]([O-])=O	62-23-7	1.93	2.18	2.29	2.13	Train
137	CC1(C)NC(=O)N(C2=CC(=C(C=C2)[N+]([O-])=O)C(F)(F)F)C1=O	63612-50-0	3.21	3.23	3.11	3.31	Train
138	[O-][N+](=O)C1=CC=C(OC2=C(Cl)C=C(Cl)C=C2Cl)C=C1	1836-77-7	1.47	1.77	2.06	1.57	Train
139	FC(F)(F)C1=CC(=C(OC2=CC=C(C=C2)[N](=O)=O)C=C1)Cl	42874-01-1	2.50	2.37	2.40	2.40	Train
140	CCOC1=CC(=CC=C1[N+]([O-])=O)OC2=CC=C(C=C2Cl)C(F)(F)F	42874-03-3	1.86	2.37	2.37	2.46	Train
141	[O-][N+](=O)C1=CC=C(OC2=CC=C(C=C2[N+]([O-])=O)C(F)(F)F)C=C1	15457-05-3	1.56	2.02	1.93	2.31	Train
142	[O-][N+](=O)C1=CC(=C(NC2=C(Cl)C=CC(=C2)C(F)(F)F)C(=C1)C(F)(F)F)[N+]([O-])=O	62441-54-7	3.66	3.85	3.76	3.76	Train
143	CN(C1=C(Cl)C=C(Cl)C=C1)C2=C(C=C(C=C2[N+]([O-])=O)[N+]([O-])=O)C(F)(F)F	84529-63-5	4.96	5.04	5.06	4.55	Train
144	CN(C1=CC=C(Cl)C=C1Br)C2=C(C=C(C=C2C(F)(F)F)[N+]([O-])=O)[N+]([O-])=O	84529-59-9	5.00	4.92	4.69	4.81	Train
145	CCO[P](=S)(OCC)OC1=CC(=C(Cl)C=C1)[N+]([O-])=O	84197-35-3	3.51	3.69	3.61	3.61	Train
146	CO[P](=S)(OC)OC1=CC=C(C=C1Cl)[N+]([O-])=O	2463-84-5	3.02	3.47	3.34	3.51	Train
147	C[P](=S)(OC1=CC=CC=C1)OC2=CC=C(C=C2)[N+]([O-])=O	2665-30-7	4.59	4.65	4.49	4.49	Train
148	CCN(CC)[P]1(=S)OCC2=C(O1)C=CC(=C2)[N+]([O-])=O	99300-57-9	3.30	3.54	3.35	3.66	Train
149	CN(C)[P]1(=S)OCC2=C(O1)C=CC(=C2)[N+]([O-])=O	99300-64-8	3.34	3.14	3.24	2.93	Train
150	CCO[P](=S)(CC)OC1=CC=C(C=C1Cl)[N](=O)=O	3563-52-8	4.13	4.15	4.03	4.03	Train
151	CCO[P](=S)(CC)OC1=CC=C(C(=C1)C)[N+]([O-])=O	18313-91-2	4.46	4.33	4.05	4.43	Test

152	CCO[P](=S)(OCC)OC1=C(Cl)C=C(C=C1)[N+]([O-])=O	6012-87-9	4.51	4.50	4.50	4.13	Train
153	CC[P](=S)(OC)OC1=CC=C(C=C1Cl)[N](=O)=O	2917-21-7	4.11	3.82	3.80	3.64	Test
154	CCO[P](=S)(OCC)OC1=CC(=C(C=C1)[N+]([O-])=O)Cl	3070-19-7	3.81	4.34	4.41	3.91	Train
155	CCCO[P](C)(=S)OC1=CC=C(C=C1)[N](=O)=O	1085-34-3	4.96	5.07	4.86	4.89	Train
156	CO[P](=S)(OC(C)C)OC1=CC=C(C=C1)[N](=O)=O	13955-12-9	4.76	4.74	4.66	4.45	Train
157	CO[P](=S)(OC(C)C)OC1=CC(=C(C=C1)[N+]([O-])=O)C	513385	4.09	4.26	4.18	4.08	Train
158	CCO[P](=S)(OCC)OC1=CC(=C(C=C1)[N](=O)=O)C	2425-15-2	4.49	4.78	4.59	4.63	Train
159	CCO[P](=S)(OCC)OC1=C(Cl)C=C(Cl)C(=C1)[N+]([O-])=O	84197-36-4	3.56	3.52	3.46	3.46	Train
160	NC1=NC=C(\C=C\C2=CC=C(O2)[N+]([O-])=O)N=N1	556-12-7	2.97	2.54	2.39	2.81	Train
161	CC1=NC(=C[N]1C2=CC=C(C=C2)[N+]([O-])=O)[N+]([O-])=O	21721-92-6	1.71	1.65	1.81	1.64	Train
162	NC1=NN=C(S1)\C=C\C2=CC=C(O2)[N](=O)=O	1020-76-4	2.10	2.02	2.00	2.20	Train
163	COC(=O)C1=C(C)NC(=C(C1C2=CC=CC=C2[N+]([O-])=O)C(=O)OCC(C)C)C	63675-72-9	2.49	2.35	2.39	2.39	Train
164	CCOC(=O)C1=C(C)NC(=C(C1C2=CC=CC(=C2)[N+]([O-])=O)C(=O)OC)C	39562-70-4	1.37	1.74	2.07	1.47	Train
165	CNC(=O)N1C(=O)CN=C(C2=CC=CC=C2)C3=CC(=CC=C13)[N](=O)=O	27016-91-7	2.17	2.13	2.27	2.07	Train
166	O=[N](=O)C1=CC=C(SC2=CC=C(C=C2)[N](=O)=O)C=C1	1223-31-0	2.27	2.24	2.26	2.32	Train
167	CCC(C)C1=CC(=CC(=C1OC(C)=O)[N+]([O-])=O)[N+]([O-])=O	2813-95-8	3.67	3.74	3.43	3.99	Test
169	CCCN(CCC)C1=C(C=C(C=C1[N+]([O-])=O)C(C)C)[N+]([O-])=O	33820-53-0	1.79	1.97	2.15	1.89	Train
170	CCCN(CCC)C1=C(C=C(C=C1[N+]([O-])=O)C(C)=O)[N+]([O-])=O	52129-71-2	2.62	2.44	2.52	2.40	Train
171	CCC(C)C1=C(OC(=O)OC2=C(C=C(C=C2)[N+]([O-])=O)[N+]([O-	61614-62-8	3.62	3.50	3.52	3.32	Train

	])=O)C(=CC(=C1)[N+]([O-])=O)[N+]([O-])=O						
172	CC(=O)OC1=C(C=C(C(=C1[N+]([O-])=O)C)[N+]([O-])=O)C(C)(C)C	214405	3.85	3.84	3.75	3.75	Train
173	CC(=O)OC1=C(C=C(C=C1C(C)(C)C)[N+]([O-])=O)[N+]([O-])=O	3204-27-1	3.66	3.85	3.76	3.76	Train
174	CCC(CC)NC1=C(C(=C(C)C=C1[N+]([O-])=O)C)[N+]([O-])=O	40487-42-1	2.43	2.31	2.41	2.25	Test
175	CCCN(CCC)C1=C(C=C(C)C=C1[N+]([O-])=O)[N+]([O-])=O	6794	1.89	2.29	2.55	2.02	Train
176	CCC(C)NC1=C(C=C(C=C1[N+]([O-])=O)C(C)(C)C)[N+]([O-])=O	33629-47-9	2.07	2.29	2.10	2.66	Train
177	CN(CCO)C1=CC=C(C=C1)N=NC2=C(Cl)C=C(C=C2Cl)[N](=O)=O	6232-56-0	2.12	2.17	2.22	2.22	Train
178	ClC1=CC(=CC(=C1N=NC2=CC=C(C=C2)N3CC[S](=O)(=O)CC3)Cl)[N](=O)=O	17741-62-7	2.13	2.79	3.31	2.03	Train
179	CCCOCCOC(=O)C1=C(C)NC(=C(C1C2=CC=CC(=C2)[N+]([O-])=O)C(=O)OCCOCCC)C	22609-73-0	2.52	2.61	2.62	2.62	Train
180	CCCCCCCCCOC(=O)C1=CNC=C(C1C2=CC=CC=C2[N+]([O-])=O)C(=O)NC3CCCC3	110263-73-5	4.68	3.78	3.79	3.55	Train
181	COCCOC(=O)C1=C(C)NC(=C(C1C2=CC(=CC=C2)[N+]([O-])=O)C(=O)OC\C=C\C3=CC=CC=C3)C	132203-70-4	2.05	2.09	2.15	2.15	Train
182	OC1=C(CNCCCl)C=C(C=C1)[N+]([O-])=O	56538-00-2	3.96	3.47	3.41	3.41	Test
183	OC(C(COC(=O)CCC(O)=O)NC(=O)C(Cl)Cl)C1=CC=C(C=C1)[N](=O)=O	3544-94-3	1.71	1.68	1.81	1.72	Train
184	OC(C1COC(=N1)C(Cl)Cl)C2=CC=C(C=C2)[N+]([O-])=O	76738-28-8	1.71	1.81	1.81	2.02	Train
185	OC1=C(C=C(Cl)C=C1[N+]([O-])=O)C2=C(O)C(=CC(=C2)Cl)[N+]([O-])=O	10331-57-4	4.54	4.68	4.44	4.64	Train
186	CCOC1=CC=C(C=C1CNCCCl)[N+]([O-])=O	56538-02-4	4.01	3.96	3.70	4.08	Train
187	COC1=CC=C(C=C1CNCCCl)[N+]([O-])=O	56538-01-3	3.94	3.94	3.84	3.84	Train

188	CO[P](=S)(OC)OC1=CC(=C(C(=C1)Cl)[N+]([O-])=O)Cl	50590-01-7	2.65	2.55	2.55	2.60	Train
189	CCO[P](=S)(OC)OC1=CC=C(C=C1)[N+]([O-])=O	2591-57-3	4.99	5.02	4.85	4.80	Test
190	CCO[P](=S)(OCC)OC1=CC=C(C=C1)[N+]([O-])=O	56-38-2	5.16	4.89	4.60	4.87	Train
191	CO[P](=S)(OC)OC1=CC=C(Cl)C(=C1)[N+]([O-])=O	5826-76-6	2.77	2.88	2.87	2.87	Train
192	[O-][N+](=O)c1cc(c(O)c(c1)[N+]([O-])=O)C1CCCCC1	131-89-5	3.61	3.52	3.56	3.31	Train
193	Ic1cc(cc(I)c1O)[N+]([O-])=O	305-85-1	3.50	3.46	3.41	3.41	Train
194	CC(C)(C)c1cc(cc(c1O)C(C)(C)C)[N+]([O-])=O	728-40-5	3.00	3.13	3.10	3.10	Train
195	S=P(C)(Oc1cc(c(cc1)[N+]([O-])=O)C(F)(F)F)OCC(=C)Cl	740-20-5	3.27	3.30	3.17	3.37	Train
196	S=P(Oc1ccc(cc1)[N+]([O-])=O)(CCl)OCC	2425-19-6	3.36	4.02	3.90	3.93	Train
197	O=C(C)Oc1c(cc(cc1[N+]([O-])=O)[N+]([O-])=O)C(C)CC	2813-95-8	3.67	3.62	3.40	3.77	Train
198	O=[N+]([O-])c1ccc(CSP(=S)(OCC)OCC)cc1	13286-49-2	3.50	3.68	3.61	3.61	Train
199	CC(=O)Nc1ccc(Cc2ccccn2)c(c1)[N+]([O-])=O	24733-81-1	3.13	3.10	3.23	2.85	Train
200	[O-][N+](=O)c1ccccc1C1OC(CO1)CCl	53460-81-4	3.57	3.25	3.47	2.82	Train
201	CCCCCC(=O)Nc1ccc(Cc2ccccn2)c(c1)[N+]([O-])=O	63233-53-4	3.51	3.60	3.62	3.41	Train
202	CCCCCCCC(=O)Nc1ccc(Cc2ccccn2)c(c1)[N+]([O-])=O	63233-54-5	3.55	3.66	3.62	3.54	Train
203	CCCCCCCCCC(=O)Nc1ccc(Cc2ccccn2)c(c1)[N+]([O-])=O	63233-55-6	3.58	3.72	3.68	3.57	Train
204	O=[N+]([O-])c1ccc(cc1Cl)OP(=O)(OC)SC	90110-57-9	3.47	3.56	3.57	3.37	Train

Table S2. True external test set for evaluating the ensemble QSAR model

ID	SMILES	CAS-ID	-logLD$_{50}$ (rat toxicity)	Ensemble prediction	SVR_A prediction	SVR_B prediction	Set
1	Oc1c(cccc1[N+](=O)[O-])[N+](=O)[O-]	573-56-8	3.68	3.45	3.40	3.38	External set
2	CN(N=O)c1ccc(cc1)N=O	99-80-9	2.08	2.00	2.32	1.71	External set
3	CCOC(=O)c1ccc(cc1)[N+](=O)[O-]	99-77-4	1.86	1.87	1.85	2.08	External set
4	CCOP(=O)(Oc1ccc(cc1)[N+](=O)[O-])SCC	597-88-6	4.21	4.41	3.88	4.85	External set
5	COC(=O)C1=C(C)NC(=C(C1c2cccc(c2)[N+](=O)[O-])C(=O)OC\C=C\c3ccccc3)C	99522-79-9	2.18	1.86	1.85	2.05	External set
6	[O-][N+](=O)c1ccn(=O)c2ccccc12	56-57-5	3.67	3.21	3.04	3.37	External set
7	[O-][N+](=O)c1oc(\C=N\N2CC(=O)NC2=O)cc1	67-20-9	2.59	2.71	3.01	2.29	External set
